# Case report: *De novo* variant of *SETD1A* causes infantile epileptic spasms syndrome

**DOI:** 10.3389/fneur.2023.1278035

**Published:** 2023-10-19

**Authors:** Mingping Lan, Yanjuan Wang, Sixiu Li, Lili Zhao, Ping Liu, Wenguang Hu

**Affiliations:** Department of Pediatric Neurology, Chengdu Women's and Children's Central Hospital, School of Medicine, University of Electronic Science and Technology of China, Chengdu, China

**Keywords:** *SETD1A gene*, infantile epileptic spasms syndrome, whole exome sequencing, case report, review

## Abstract

Infantile epileptic spasms syndrome (IESS) is one of the most common epileptic encephalopathies of infancy, with typical clinical features defined by a triad of epileptic spasms, hypsarrhythmia, and developmental delay. Genetic factors are important causes of IESS. The *SETD1A* (SET Domain Containing 1A) gene encodes a histone lysine methyltransferase that activates gene transcription through histone H3 lysine K4 methylation. Mutations in the *SETD1A* gene have been associated with schizophrenia, and some have been reported to cause seizures. Herein, we report a case of IESS caused by a *SETD1A* gene mutation. Video electroencephalography showed hypsarrhythmia. No specific findings were obtained after brain MRI and metabolic work-up. The seizures disappeared after treatment with adrenocorticotropic hormone, vitamin B6, and valproic acid during hospitalization. Genetic testing revealed that the child had a variant (NM_014712.3:c.3005_3,006 delAG, p.Glu1002Glyfs*20) in exon 12 of the *SETD1A* gene, representing a *de novo* mutation. There have been no previous reports on the *SETD1A* gene causing infantile spasms. We also summarize the existing literature on *SETD1A* gene–related epilepsy to provide a reference for clinical diagnosis and treatment.

## Introduction

Epilepsy is a chronic non-communicable neurological condition affecting individuals of all age groups. It is one of the most common and prevalent neurological disorders, with approximately 50 million people living with epilepsy worldwide. Research suggests that with proper diagnosis and treatment, up to 70% of people with epilepsy could become seizure-free. Despite numerous studies, in nearly 50% of cases, the exact etiology of the disease remains elusive. The causative factors are classified into six categories, including structural, genetic, infectious, metabolic, immune, and unknown cause (1).

Infantile epileptic spasms syndrome (IESS) is one of the most common epileptic encephalopathies of infancy. First described by William James West in 1841 (which led later on to the naming of the condition as West syndrome), IESS is estimated to have an incidence of 2–4 per 10,000 surviving infants (2). Typical clinical features include a triad of epileptic spasms, hypsarrhythmia, and developmental delay or regression; however, some IESS cases lack one of these three criteria. The vast majority of IESS occurs in the first year of life, and the peak age of onset is 3–12 months. The prognosis is usually poor and may be accompanied by varying degrees of developmental delay (3). Genetic factors are important causes of IESS, and with the development of next-generation sequencing (NGS), several gene mutations have been linked with the disease. The genes identified include *STXBP1*, *KCNQ2*, *SCN2A*, *SCN8A*, *ALG13*, *GABRA1*, *DNM1*, *GNAO1*, *GRIN1*, *PTEN*, *TUBB2A*, and *KCNT1* (4).

*SETD1A* (SET Domain Containing 1A), a member of the SET subfamily of protein methyltransferases, is located at 16p11.2 and contains highly conserved SET and post-SET domains at the C-terminus. It encodes a histone lysine methyltransferase that can regulate gene expression and the cell growth cycle. *SETD1A* interacts with other proteins to form a highly conserved complex called COMPASS (Complex Proteins Associated with SET1), which determines *SETD1A* methylation activity (5). Histone lysine methyltransferases function mainly by transferring methyl groups to lysine residues of histones, primarily at H3K4, H3K9, H3K27, H3K36, H3K79, and H4K20. *SETD1A* leads to gene activation through methylation of H3K4, which is mainly distributed in gene promoter regions and plays an important role in transcription initiation and elongation. It has been reported that some children with mutations in the *SETD1A* gene present seizures (6–8); however, there have been no reports directly linking *SETD1A* alterations with IESS. Here, we report a case of IESS caused by a *SETD1A* gene variant and summarize the literature to provide a reference for clinical diagnosis and treatment.

## Patient information

**Figure 1:**
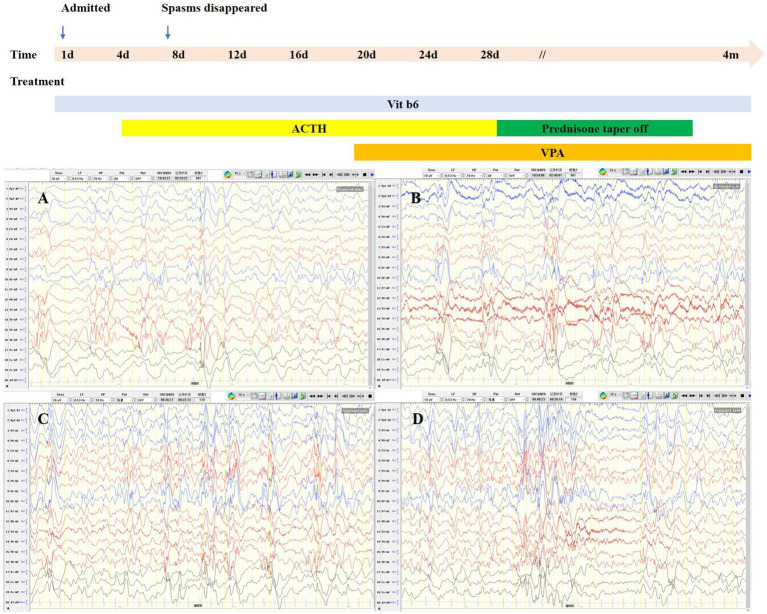
Interictal VEEG of the patient. **(A,B)**: awakening; **(C,D)**: sleeping.

**Figure 2:**
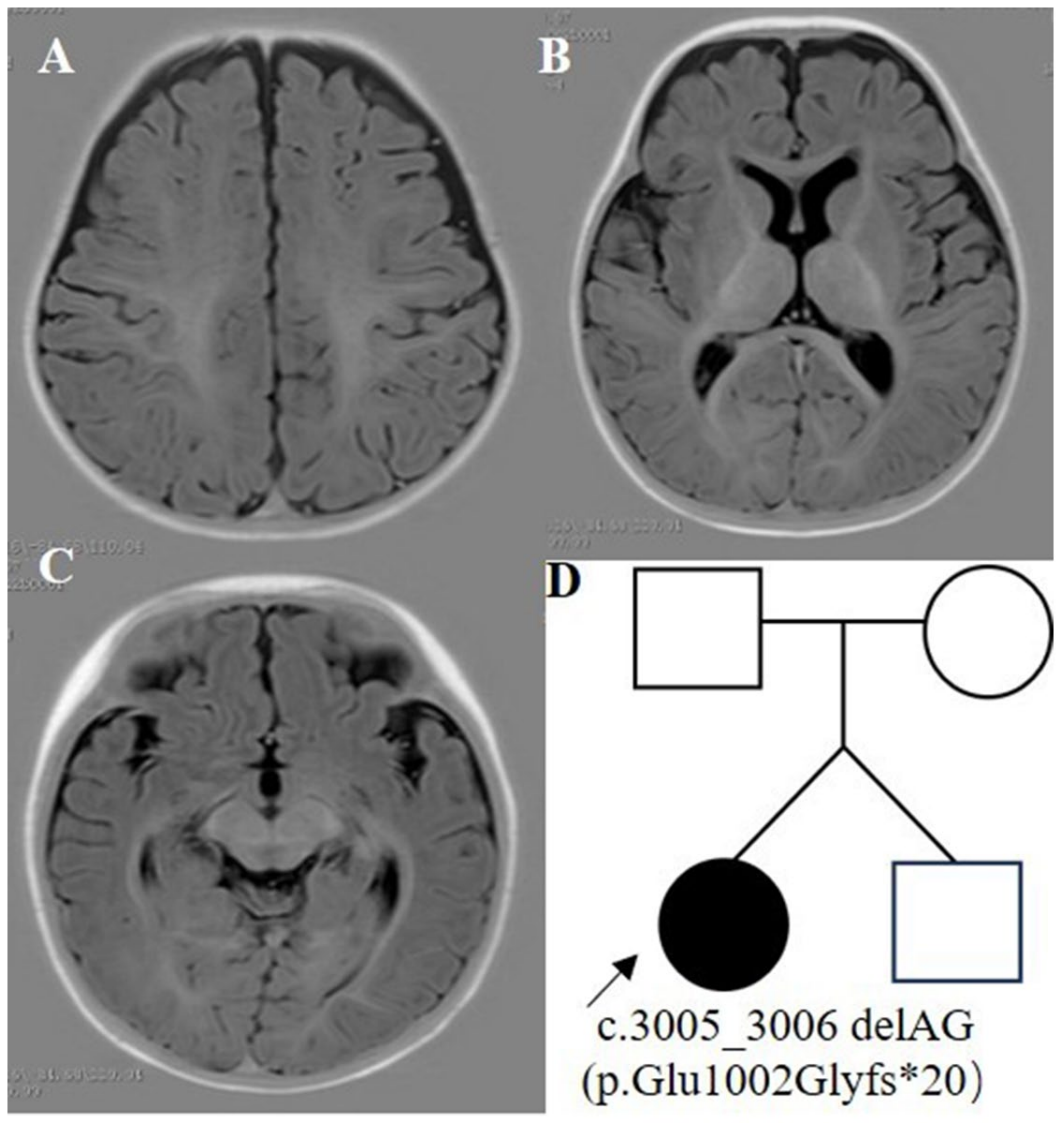
Cranial MRI and family pedigree. **(A–C)** Cranial MRI. **(D)** Family pedigree.

The proband was a 6-month-old female, one of the twins of healthy, nonconsanguineous parents. The girl was born at full term and with a history of mild asphyxia. She had a birth weight of 2,000 g and no history of feeding problems. There was no history of genetic diseases in her family and the mother denied a history of teratogenic agents and radiation exposure during the pregnancy. After the neonatal period, she appeared to have a mild developmental delay; she could raise her head at 3 months but could not roll over at 6 months. Her twin brother was unremarkable. Seizures developed at 6 months of age and were characterized by epileptic spasms. The physical examination was unremarkable. Laboratory studies, including complete blood counts, liver and renal function, electrolytes, lactate, ammonia, and metabolic screening were normal. Her video-electroencephalogram (VEEG) showed hypsarrhythmia (Figure 1), and cranial MRI was normal (Figure 2).

## Genetic analysis

After informed consent was obtained from the parents, peripheral venous blood samples (3 mL) were collected from the child, her brother, and her parents and anticoagulated with ethylenediaminetetraacetic acid (EDTA).

DNA library preparation was performed following Illumina protocols and included end repair, adapter ligation, and PCR enrichment. The amplified DNA was then captured using an IDT xGen Exome Research Panel (Cipher Gene, Beijing, China). The raw data obtained were converted from .bcl files to .fastq files using bcl2fastq software. The reads were then aligned to the human reference genome GRCh38/hg38 using BWA, SAMtools, and Picard software. The GATK software suite was employed for local realignment and duplicate sequence removal. Variant annotations were conducted using ANNOVAR software. The principles for screening pathogenic variants were as follows: (1) screening for variants in the exonic regions, including non-synonymous mutations; (2) identification of variants that are absent or with a frequency less than 5% in databases such as ExAC, 1,000 Genomes, and gnomAD, which contain data from healthy individuals; (3) evaluation of pathogenic variants based on various databases, including dbSNP, OMIM, HGMD, and ClinVar; and (4) verification by Sanger sequencing of pathogenic variants associated with clinical phenotypes detected by NGS. Pathogenicity of mutations was assessed following the American College of Medical Genetics and Genomics (ACMG) guidelines.

## Genetic results

**Figure 3:**
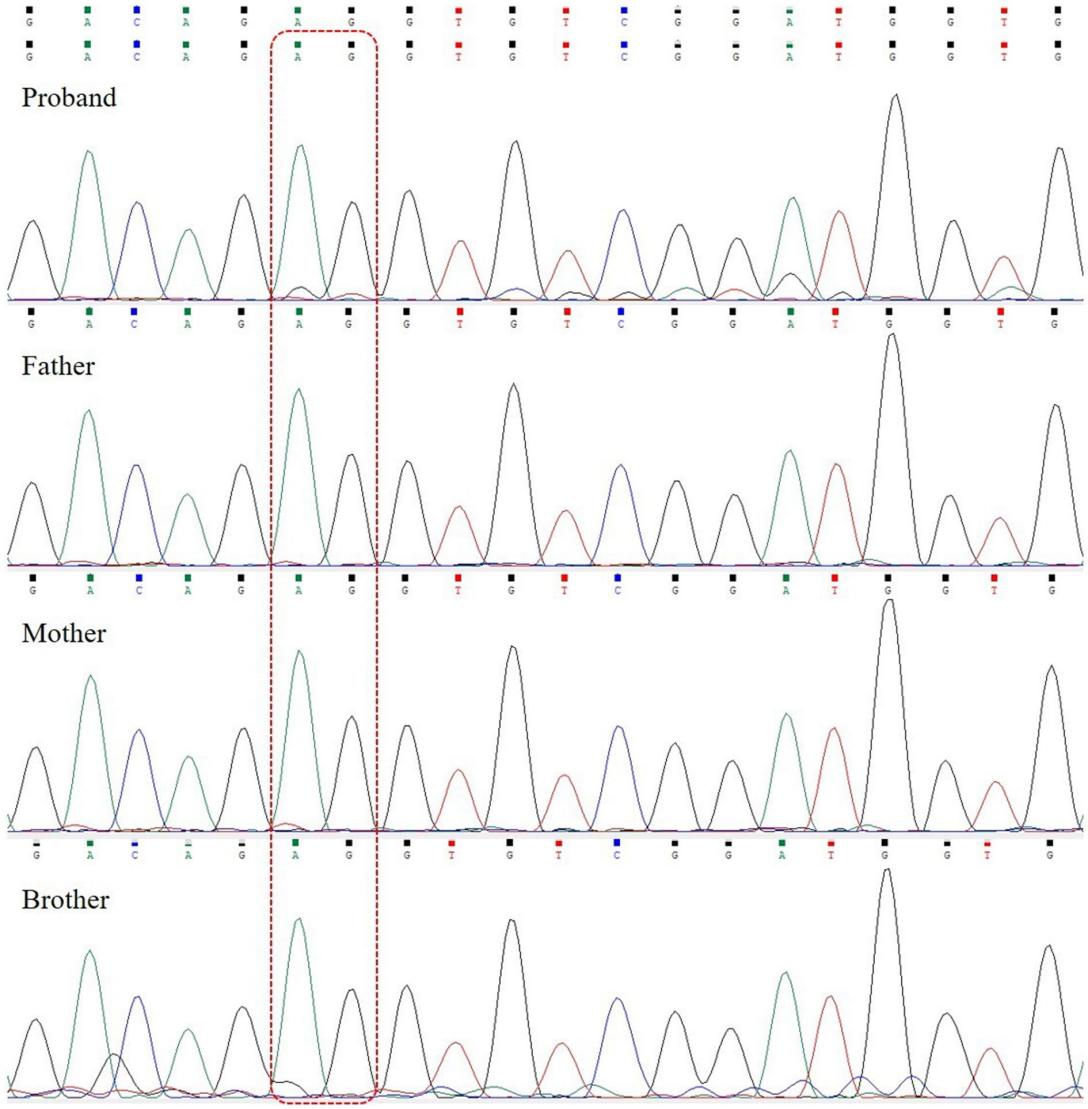
*SETD1A* gene sequencing of the patient and her family. The patient carried a heterozygous mutation (c.3005_3,006 delAG); the father, mother, and brother are wild-type. The dashed line box indicates the location of variants.

Whole exome sequencing (WES) revealed a heterozygous variant c.3005_3,006 deletion (NM_014712.3:c.3005_3,006 delAG, p.Glu1002Glyfs*20) in exon 12 of the *SETD1A* gene (Figure 3). This variant was detected as a mosaic mutation, and a frequency of 11.8% was revealed for the mutant allele at 161X coverage by NGS. Sanger sequencing confirmed that the parents and brother were wild-type. This variant could result in a frameshift mutation, and it was absent in major genomic databases (i.e., gnomAD, ExAC, and 1,000 Genomes). It was classified as a pathogenic variant (PVS1 + PS2_Supporting+PM2_Supporting) according to ACMG guidelines.

## Diagnostic assessment

Considering her age, clinical manifestations, and VEEG features, we considered that she had IESS, and empirical treatment with adrenocorticotropic hormone (ACTH, 2 U/kg/d) and vitamin B6 (15 mg/kg/d) was performed during hospitalization. The epileptic spasms disappeared after 3 days of ACTH administration. Considering the good response, we used ACTH for a total of 4 weeks and then switched to oral prednisone. Since most patients do not become seizure-free with ACTH alone, we also added valproic acid after 2 weeks of ACTH treatment. To date, the girl has been followed up for 4 months, prednisone has been discontinued, and she remains seizure-free. The child is able to sit unsupported, albeit unstably, and can crawl. She can grasp and babble. Reexamination of VEEG showed disappearance of hypsarrhythmia and slow waves in bilateral temporal areas.

## Literature review

PubMed and Google Scholar were searched for articles up to 1 August 2023. The following terms were used in the literature search: “*SETD1A*” or “SET Domain Containing 1A.” Articles with reports of seizures were screened. The clinical and genetic characteristics of patients are summarized in Table 1.

**Table 1:** Clinical features of *SETD1A* patients with seizures.

Gender	Variant	Inheritance	Onset	Seizure type	EEG	MRI	ASM	Age at follow-up	Development
F	Exon 10 c.2737C>T p.R913C	Inherited^a^	2 d	Ct	Some sharp waves and sharp-slow-waves	Normal	PB	1 y and 3 m	Normal
F	Exon 6 c.806A>G p.Q269R	*De novo*	2 y	Ct	Bilateral spike waves and sharp-and-slow-waves	Normal	NA	2 y and 6 m	Normal DQ = 110 MI = 107
M	Exon 14 c.4105G>A p.G1369R	*De novo*	9 m	Ct	Multiple spike-and-slow-waves and slow-spike-and-waves, especially when sleeping	Cerebral dysplasia	NA	1 y and 9 m	Developmental delay
M	Exon 14 c.4175G>A p.R1392H	*De novo*	1 d	Ct	Bilateral sharp waves and spike-and-waves	Subdural hemorrhage	NA	NA	NA
F	Exon 8 c.2120_2121insA (p.Gly708Argfs*117)	*De novo*	3 y	GTCS	Mild background slowing	Bilateral white matter dysplasia and ventriculomegaly	LEV^b^	7 y and 6 m	Developmental delay WISC: 65
F	Exon 12 c.3005–3,006 del (p.Glu1002Glyfs*20)	*De novo*	6 m	ES	Hypsarrhythmia	Normal	ACTH,VPA,Vit B6	9 m	Developmental delay

PB, phenobarbital; LEV, levetiracetam; WISC, Wechsler Intelligence Scale for Children; VPA, Valproic acid; Ct, Clonic and tonic; GTCS, Generalized tonic–clonic; ES, Epileptic spasms; NA, no data; data were not available for the other 6 patients.

a Father, grandfather, and grandmother had convulsions, but the gene was not detected.

b Seizure-free by LEV but relapse 4 years later.

## Discussion

We report a case of IESS caused by a heterozygous variant (NM_014712.3:c.3005_3,006 delAG, p.Glu1002Glyfs*20) in exon 12 of the *SETD1A* gene. To the best of our knowledge, this variant has not been previously reported in the literature and this is the first report of a *SETD1A* gene mutation causing IESS.

Histones are the major protein components of chromatin and play an important role in gene regulation. Methylation is a common post-translational modification of histones and can occur on lysine and arginine residues. Loss-of-function (LOF) variants in methyltransferases are associated with developmental disorders such as Kabuki syndrome, autism, Kleefstra syndrome, O’Donnell-Luria-Rodan syndrome, and some intellectual disabilities (7, 9, 10). The SET family is divided into seven subfamilies, namely, Suv, Ash, Trx, E (z), PRDM, SMYD, and SETD (11). The SETD family contains 10 members, including *SETD1A*, *SETD1B*, and *SETD2*-*9*. SETD has methyltransferase activity and can regulate gene expression and the cell growth cycle (12). *SETD1A*, also known as *KMT2F*, mediates H3K4 methylation, which is regarded as a mark of transcriptional activation and is important to prevent DNA damage at stalled replication forks (13).

*SETD1A* has previously been associated with schizophrenia. After analyzing WES data, Singh and colleagues found that 10 out of 7,776 schizophrenia families carried *SETD1A* variants and concluded that LOF mutations in *SETD1A* are a risk factor for the condition (8). In addition, a study of 231 patients with schizophrenia detected two patients with *SETD1A de novo* mutations, suggesting that *SETD1A* variants may contribute to the risk of neurodevelopmental disorders (14).

However, so far, there are few reports about *SETD1A*-related epilepsy, and none of those described *SETD1A*-related IESS. Upon searching the databases, seizures were reported in 11 patients with *SETD1A* gene mutations (6–8, 15, 16); after excluding cases without detailed data, the clinical data of the remaining cases are summarized in Table 1. Of the six patients reviewed, four had missense mutations, two had frameshift mutations, and five had *de novo* mutations. Two patients had seizure onset in the neonatal period, two patients had seizure onset in infancy, and two had seizure onset after 1 year of age. The seizures were tonic–clonic in five cases and epileptic spasms only in our case. Except for our patient, who showed hypsarrhythmia on EEG, the other cases were nonspecific, with background slowing, sharp waves, or spike-and-waves. Brain MRI showed that three were normal, two had cerebral atrophy, and one had subdural hemorrhage. The treatment of *SETD1A*-related epilepsy is still not well established, and antiseizure medications (ASMs) were used in three cases (including our own). One patient was treated with phenobarbital and seizure frequency was reduced; however, no information was provided regarding subsequent episodes. Another patient achieved seizure-free status after receiving levetiracetam. However, she recurred 4 years later, with no obvious abnormalities on repeated VEEG at that time. Our patient was treated empirically with vitamin B6 on admission and seizure remission was achieved after 3 days of ACTH infusion. Considering that most children with IESS are drug-resistant and can rarely be controlled with a single ASM, we later added valproic acid (VPA). Since vitamin B6 had been used for several days, but spasms persisted until ACTH was added and genetic testing did not suggest alterations in a vitamin B6-related gene (e.g., *ALDH7A1*), we do not believe that vitamin B6 deficiency was involved. In addition, our case is responding well to ASMs, which may be due to the child’s mosaicism. Currently, the girl is 10 months old and remains seizure-free despite the discontinuation of corticosteroids.

Three out of the six patients reviewed had developmental delay, which was mild in one case. Our patient cannot currently sit stably and her parents refused to complete the developmental assessment; therefore, we could not obtain an accurate developmental score. Since a short time has lapsed since she was diagnosed and treated, a longer follow-up is needed to verify seizure control and to assess physical and cognitive development. At the same time, related studies are needed to arrive at an optimal treatment.

In conclusion, the variant NM_014712.3:c.3005_3,006 delAG of the *SETD1A* gene was identified as the causative factor of IESS in a 6-month-old girl. This is the first report of IESS caused by a *SETD1A* gene variant, along with a concise account of the diagnosis, treatment, and follow-up of the child. Our findings further expand the mutational and phenotypic spectra of the *SETD1A* gene and provide a reference for clinical diagnosis and treatment of IESS.

## Data availability statement

The datasets presented in this article are not readily available because of ethical and privacy restrictions. Requests to access the datasets should be directed to the corresponding author.

## Ethics statement

The studies involving humans were approved by the Ethics Committee of Chengdu Women’s and Children’s Central Hospital. The studies were conducted in accordance with the local legislation and institutional requirements. Written informed consent for participation in this study was provided by the participants’ legal guardians/next of kin. Written informed consent was obtained from the individual(s), and minor(s)’ legal guardian/next of kin, for the publication of any potentially identifiable images or data included in this article.

## Author contributions

ML: Writing – original draft, Data curation. YW: Data curation, Writing – review & editing. SL: Data curation, Writing – review & editing. LZ: Writing – review & editing. PL: Writing – review & editing. WH: Supervision, Writing – review & editing.
